# Supplementary material for: KRT6A and KRT17 Mark Distinct Stem Cell Populations in the Adult Palpebral Conjunctiva and Meibomian Gland
Source: Cells. 2025 Dec 12;14(24):1979. doi: 10.3390/cells14241979 (PMC12732064; doi:10.3390/cells14241979)
Supplement: Supplementary file 1 [file cells-14-01979-s001.zip › cells-3993596-supplementary figures.pdf]

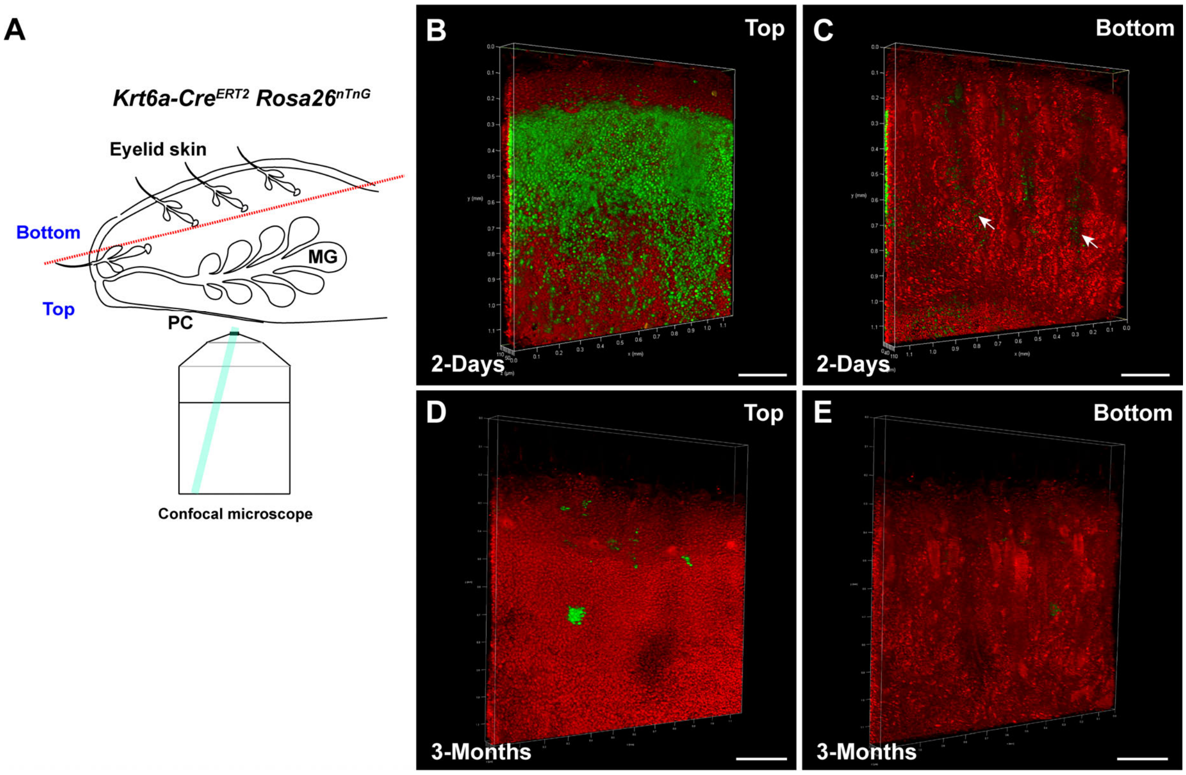

Figure S1. KRT6A marks self-renewing SCs in the PC but not MG. (A) Schematic depicting the experimental setup for whole-mount confocal imaging. (B, C) Representative 3D whole-mount fluorescent images show that $Krt6a^{GFP}$ cells are broadly present within the PC (B) and sporadically distributed in the MGs (C, white arrows) following 2 days of tamoxifen administration. (D, E) 3D whole-mount fluorescent images indicate that KRT6-expressing cells form clones in the PC (D) but not within MGs (E) in mice subjected to 3 months of lineage tracing. N=4 mice were analyzed at each time point. Scale bars: 200 µm.

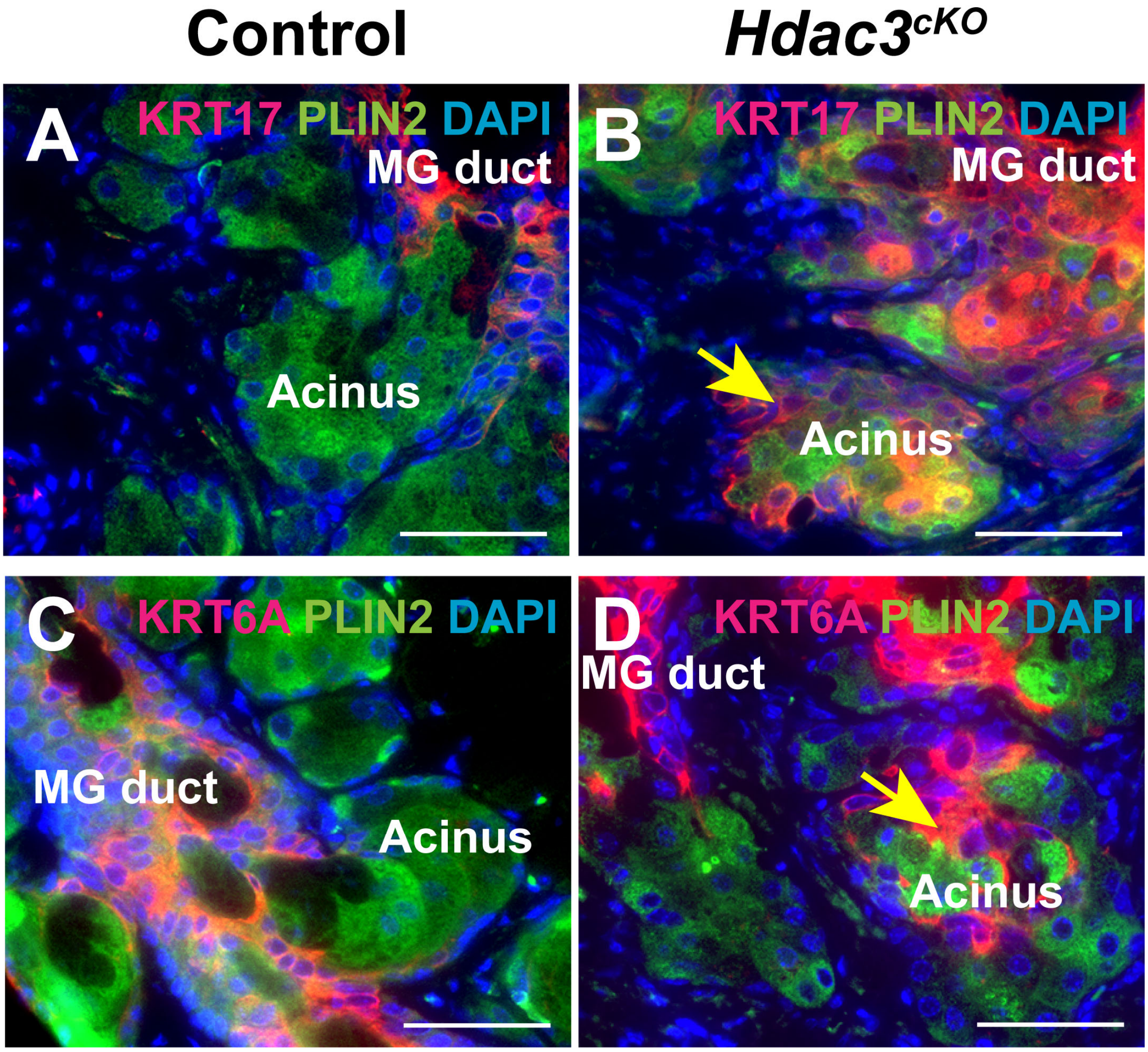

Figure S2. HDAC3 represses KRT17 and KRT6A expression in the MG acinus. (A, B) IF shows that expression of KRT17 is primarily detected in the duct of control MGs (A) but expands to the PLIN2+ acinus in *Hdac3*-deficient MGs (B, yellow arrow). (C, D) IF indicates that KRT6A expression is enriched in control MGs (C) but exhibits ectopic expression in the acinus of *Hdac3*-deficient MGs (D, yellow arrow). N=4 mice per genotype were analyzed. Scale bars: 25 μm.

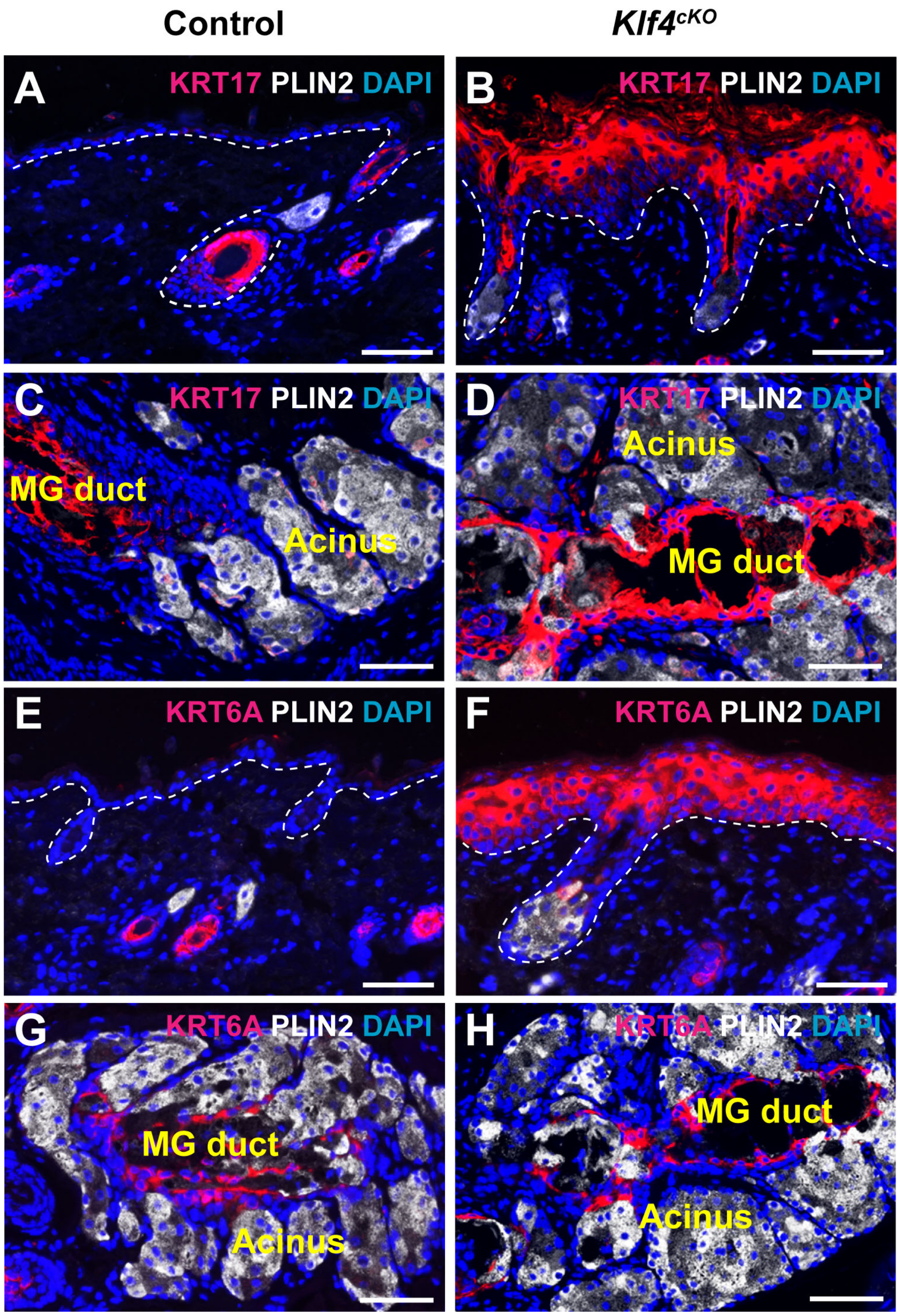

Figure S3. KLF4 negatively regulates KRT17 and KRT6A in adult eyelid interfollicular epidermis. (A, B) IF shows that KRT17 is not expressed in control eyelid interfollicular epidermis (A) but is ectopically expressed in *Klf4*-deficient eyelid interfollicular epidermis (B). (C, D) IF indicates that KRT17 exhibits similar expression in control (C) and *Klf4*-deficient MGs (D). (E, F) IF reveals that KRT6A expression is absent in control eyelid interfollicular epidermis (E) and that *Klf4*-deficient eyelid interfollicular epidermis exhibits strong KRT6A expression (F). (G, H). IF detects comparable KRT6A expression between control (G) and *Klf4*-deficient (H) MGs. N=3 mice per genotype were analyzed. Scale bars: 25 μm.
